# Characteristics of platelet-associated parameters and their predictive values in Chinese patients with affective disorders

**DOI:** 10.1186/s12888-022-03775-9

**Published:** 2022-02-25

**Authors:** Yanyan Wei, Junhui Feng, Jinbao Ma, Dongning Chen, Haiting Xu, Lu Yin, Jingxu Chen

**Affiliations:** 1 Beijing Hui-Long-Guan Hospital, Peking University, Beijing, 100096 China; 2 Jining Psychiatric Hospital, Jidai Road 1#, Jining, 272000 Shandong, China; 3 Beijing Tongren Hospital, Dongjiaomin Road 1#, Beijing, 100000 China

**Keywords:** Major depressive disorder, Bipolar disorder, Platelet-associated parameters, Mean platelet volume, Systemic immune-inflammation index

## Abstract

**Objective:**

Platelets are increasingly considered to play an important role in inflammation and are being regarded as a putative bridge linking mental diseases and inflammatory response. Platelet-associated haematological parameters including mean platelet volume (MPV), platelet distribution width (PDW), plateletcrit (PCT), systemic immune-inflammation index (SII), platelet to lymphocyte ratio (PLR), platelet to albumin ratio (PAR) and red blood cell distribution width (RDW) to platelet ratio (RPR), have been recently investigated as simple, easily available, and inexpensive inflammatory markers. In this study, we aimed is to use large-scale clinical data to study platelet parameters in patients with affective disorders, to further investigate the predictive power of platelet parameters for major depressive disorder (MDD) and bipolar disorder (BD).

**Methods:**

The retrospective, naturalistic, cross-sectional study analysed the data of 14,007 Chinese affective disorder patients, including 4,801 patients with first-episode MDD, 4,098 patients with recurrent MDD, 3,444 patients with BD manic episodes and 1,664 patients with BD depressive episodes. Meanwhile, 6,847 healthy subjects were served as the control group. The differences in the MPV, PDW, PCT, SII, PLR, PAR, RPR and albumin among different groups were compared, and the contributing factors for the occurrence of MDD or BD were analysed.

**Results:**

There were significant differences in MPV, PDW, PCT, SII, PLR, RPR and albumin values among the study groups. In the subjects, patients experiencing BD manic episodes had the highest mean values of MPV and SII, patients experiencing BD depressive episodes had the lowest mean values of platelet counts and PAR, and patients with MDD had the highest mean values of PLR and RDW. The levels of MPV, PDW and albumin were independently correlated with MDD and BD, and they are important predictors for differentiating patients with MDD or BD from healthy controls.

**Conclusions:**

Our study demonstrated that different affective disorders have unique platelet parameter variation patterns, highlighting the role of platelet parameters and systemic inflammation in the pathophysiology of MDD and BD.

## Introduction

Affective disorders are one of the most common and debilitating mental illnesses seen in clinical practice, which severely impact mood, sleep, diet and cognitive functions, often generating incapacities to manage emotion and behaviour, in some cases, they may even drive people to suicide [1, 2]. The main types of affective disorders are major depression disorder (MDD) and bipolar disorder (BD), which seriously affect people's social function, resulting in a reduced ability to cope with daily tasks in life and work [3, 4]. Furthermore, affective disorders are one of the main causes of disability worldwide and impose a heavy burden on individuals, families and society [5]. Although considerable research on the pathogenesis of affective disorders in the past has made significant progress and has strengthened our understanding and treatment of affective disorders, there is still a subset of people who do not respond well to existing treatments [6]. In addition, approximately one-third of patients have residual symptoms and functional impairment after treatment [7]. This phenomenon reminds us that the pathophysiological mechanisms of affective disorders need to be further studied.

In recent years, the link between platelets and mental disorders has gained prominence. Platelets, also known as “thrombocytes”, are fragments of cytoplasm that are continuously produced from the mature megakaryocytes of the bone marrow and then enter the circulation [8]. Platelets share many molecular and functional characteristics with neurons [9], they both take part in the uptake and release of serotonin, which is both a central nervous neurotransmitter and a peripheral neuromodulator, playing a crucial part in the pathophysiological mechanisms of affective disorders. Previous studies have found that the abnormalities of the 5-HT receptor in platelets may suggest deficiency of the 5-HT receptor in the central nervous system [10, 11]. Furthermore, apart from its role in haemostasis and regulating 5-HT, platelets are also implicated in immune regulation [12]. Activated platelets have inflammatory functions in several physiological and pathological conditions, and studies have shown that aberrant platelet activation can mediate a series of inflammatory conditions, including atherosclerosis and acute lung injury, and they have recently been reported to be involved in the mechanisms of COVID-19 [13, 14].

Platelet parameters have been proposed as inflammatory markers in many diseases. The most commonly assessed platelet parameters included in the complete blood count (CBC) contain the platelet distribution width (PDW), mean platelet volume (MPV) and plateletcrit (PCT), which can be tested under simple laboratory conditions [15]. PDW signifies the size distribution of platelets, which is a marker of platelet anisocytosis and increases with platelet activation [16, 17]. PDW has been introduced as a marker of inflammation in diabetic nephropathy [18], irritable bowel disease [19], and coronary heart disease [20]. MPV, the most frequently studied blood-based platelet parameter, represents the average size of platelets in the blood. MPV has a positive relationship with platelet activity indicators including glycoproteins Ib receptors which correlates with platelet function and reactivity [21, 22]. MPV is associated with various inflammatory conditions including inflammatory polyps [23], diabetes mellitus [24], irritable bowel syndrome [25], malignancy [26], and obesity [27]. Plateletcrit (PCT) is a measure of the total mass of platelets as a percentage of volume in the blood and is thought to be potential marker of inflammation [28]. Furthermore, the platelet to lymphocyte ratio (PLR) and systemic immune-inflammation index (SII) based on neutrophil, lymphocyte, and platelet counts are considered to be novel inflammatory biomarkers, that were recently reported to reflect inflammatory status [29, 30]. PLR is an inflammatory predictor in diabetes mellitus [31] and irritable bowel disease [19]. Red blood cell distribution width (RDW) is an index that signifies the size of red blood cells, and the RDW to platelet ratio (RPR) is increasingly studied as a marker of chronic inflammation in physical illness and mental illness [32, 33]. RPR is considered as a marker of inflammatory burden in functional bowel diseases [34], and in mortality of intensive care population [35]. The platelet to albumin ratio (PAR) was originally studied in cancer and is considered as a clinical indicator of inflammatory status [36].

There is growing evidence that inflammation plays a key role in in the pathogenesis of affective disorders [2]. These platelet parameters derived from the CBC are simple, readily available, low-cost and reproducible biomarkers of inflammation. There have been limited studies on the use of these indicators in the diagnosis of affective disorders, and the studies are fraught with inconsistent results, which are unable to effectively illuminate the complexity of the immune system response in the different mood episodes of affective disorders.

Given the inflammatory mechanisms involved in the occurrence of mood episodes in the affective disorders, we aimed to investigate whether there were differences in platelet parameters, including the platelet count, MPV, PDW, PCT, PLR, SII, RPR and PAR, during the different mood episodes in affective disorders, including first-episode MDD, recurrent MDD, BD depressive episodes and BD manic episodes. We also analysed tthe relationship between platelet parameters and the presence of MDD and BD to further analyse the contributing factors in the occurrence of MDD and BD. To the best of our knowledge, this is the first large-scale study that has analysed the differences in these platelet parameters among patients with first-episode MDD, recurrent MDD, BD depressive episodes and BD manic episodes.

## Materials and methods

### Study population

This study was a cross-sectional, retrospective, naturalistic study that evaluated sociodemographic and haematological data extracted from the Electronic Medical Record System (EMRS) of the Beijing HuiLongGuan Hospital and Jining Psychiatric Hospital through a clinical record search tool. In present study, we focused on hospitalized patients with affective disorders, including first-episode MDD, recurrent MDD, BD depressive episodes and BD manic episodes, who were diagnosed according to the International Classification of Diseases 10th Revision (ICD-10) psychiatric diagnoses criteria. The patients were hospitalized for depressive or manic episodes in Beijing HuiLongGuan Hospital or Jining Psychiatric Hospital from January 2015 to January 2021. The analysis in the study was performed using only the first entry for each patient from the inpatient care unit. In general, the first blood test is executed the next day after admission in these centres. Therefore, we assumed that most of the patients included in our study were in the acute phase of the disease.

Patients who met the following inclusion criteria were recruited: (a) ICD-10 primary diagnosis of MDD or BD; (b) available whole blood count data and albumin levels. The exclusion criteria included (a) significant acute or severe illness such as infection, autoimmune disease, heart failure and tumor; (b) illnesses that might affect platelet levels, such as hypersplenism and bone marrow diseases. A total of 14,007 patients who met the study criteria were included in the study, including 4,801 patients with first-episode MDD, 4,098 patients with recurrent MDD, 3,444 patients with BD manic episodes and 1,664 patients with BD depressive episodes. A total of 6,847 healthy individuals with age and sex matched to the patient group were served as the control group. The healthy controls (HCs) had no history of mental illnesses, and the other exclusion criteria were the same as those in the patient group.

### Variables and laboratory tests

We extracted demographic characteristics, including age and sex, from the EMRS. Whole blood count data and biochemical test data were also extracted from the EMRS, including platelet count, MPV, PDW, PCT, neutrophil counts, lymphocyte counts, RDW, and albumin levels. Fasting venous blood was collected after 8 h of fasting in the morning, then was processed within 30 min after blood collection. Blood sample measurements were performed in the laboratory of the Beijing HuiLongGuan Hospital or Jining Psychiatric Hospital by laboratory technicians who were blinded to the diagnosis of diseases. The PLR value was measured by dividing the platelet count by the lymphocyte count, the SII level was calculated as platelet count × neutrophil count/lymphocyte count, the RPR value was calculated by deviding the RDW value to platelet value, the PAR value was measured by deviding the platelet value to albumin value.

### Ethical statement.

We informed the ethics committee of our hospital about this low-risk retrospective observational study, and the study protocol was approved by the ethics committee.

### Statistical analysis

All statistical analyses were carried out with SPSS version 21.0 and R version 4.0.3. Continuous data are presented as the mean ± standard error of the mean (SEM). The normality of the data distributions was evaluated by the Kolmogorov–Smirnov test. The data with non-normal distribution and the data with unequal variances were analysed after log or sqrt transformation. Descriptive data was analysed by the Chi-square (χ^2^). Variables with homogenous distribution were compared with t-test between two-groups or with one-way analysis of variance (ANOVA) among multiple-groups. Variables with non-homogenous distribution were compared with Mann–Whitney U Test between two-groups or with Kruskall-Wallis test among multiple-groups. To exclude the confounding influence of age and sex, analysis of covariance (ANCOVA) was applied to assess the differences in platelet parameters among different groups. A post hoc analysis that used the Bonferroni correction analysis to correct for multiple comparisons was utilized to identify the differences between groups. Correlations between platelet parameters and age were analysed using Spearman correlation analysis. A binary logistic regression analysis was utilized to detect the contributing factors for MDD or BD. Nomogram was used to predict the onset of MDD or BD using a combination of multiple indicators. Receiver operating characteristic (ROC) curve graphics were applied to analyse the sensitivity and specificity of platelet-associated parameters in differentiating between patients with MDD or BD and healthy controls. *P* < 0.05 was regarded as statistically significant.

## Results

### Comparison of variables between patients with affective disorders and healthy controls

In this study, 14,007 patients with affective disorders and 6,847 healthy controls were enrolled for the final analysis. There were no differences found in age or sex between the affective disorders group and HC group (*P* > *0.05*).

The ANCOVA analysis showed that after adjusting for age and sex, the affective disorders group showed lower platelet, neutrophil and lymphocyte counts (*P* < 0.05), and lower PDW, PCT and albumin (*P* < 0.05) than the HC group. The values of MPV, RDW and RPR in the affective disorders group were significantly higher than those in the HC group (*P* < 0.05). No significant differences were found in the PLR, SII and PAR values between the groups (*P* < 0.05). The data are shown in Table 1.

**Table 1:** Comparison of sociodemographic and laboratory variables between patiens with affective disorders and healthy controls

Variables	Affective disorders group (*n* = 14,007)	HC group (*n* = 6847)	t/F/^2^	*P*
Age(year)	42.85 ± 0.147	42.82 ± 0.157	0.144	0.886
Sex (male/female)	5844/8163	2863/3984	0.016	0.899
Platelet	238.706 ± 0.534^a^	252.994 ± 0.712	256.326	0.000
MPV	9.470 ± 0.020^a^	8.699 ± 0.014	2296.555	0.000
PDW	13.799 ± 0.020^a^	15.931 ± 0.019	4541.474	0.000
PCT	0.209 ± 0.000^a^	0.218 ± 0.000	136.260	0.000
Neutrophil	3.549 ± 0.013^a^	3.597 ± 0.014	5.061	0.024
Lymphocyte	1.912 ± 0.05^a^	1.963 ± 0.007	35.806	0.000
RDW	13.622 ± 0.014^a^	13.272 ± 0.014	257.483	0.000
Albumin	43.258 ± 0.032^a^	45.999 ± 0.033	3228.521	0.000
PLR	136.748 ± 0.479	136.981 ± 0.545	0.115	0.735
SII	494.799 ± 3.244	492.205 ± 2.910	0.254	0.614
RPR	0.061 ± 0.000^a^	0.056 ± 0.000	300.172	0.000
PAR	5.544 ± 0.000	5.520 ± 0.000	1.258	0.262

*HC* Healthy controls, *MPV* Mean platelet volume, *PDW* Platelet distribution width, *PCT* Plateletcrit, *RDW* Red blood cell distribution width, *PLR* pPlatelet to lymphocyte ratio, *SII* Systemic immune-inflammation index, *RPR* RDW to platelet ratio, *PAR* Platelet to albumin ratio, a, vs. HC group, *P* < 0.05. Values are represented as mean ± SEM

### Comparison of variables among MDD, BD and healthy controls

A total of 8,899 MDD patients and 5,108 BD patients were included in the present study. ANOVA showed that the differences in age among the MDD, BD and HC groups were stastistically significant (F = 211.320, *P* < 0.001). Post hoc analysis rendered that the BD group was younger than the MDD group and the HC group, the MDD group was older than the HC group (*P* < *0.05*) in the present study. Chi-square analysis showed that the BD group had an increased male/female ratio compared with the MDD group and the HC group, the MDD group had a decreased male/female ratio compared with the HC group (χ^2^ = 342.416, *P* < 0.001).

The ANCOVA showed that significant differences were found in the platelet, neutrophil and lymphocyte counts, and in the MPV, PDW, PCT, RDW, albumin, PLR, SII and RPR among the three groups (*P* < *0.05*), there was no difference found in the PAR among the three groups (*P* > *0.05*). Further post hoc analysis showed that compared to the HC group, the BD group had higher MPV, neutrophil, RDW, SII and RPR values (*P* < *0.05*), and lower platelet counts, PDW, PCT, albumin, PLR values (*P* < *0.05*). Compared to the HC group, the MDD group showed higher MPV, RDW, PLR and RPR values (*P* < *0.05*), and lower platelet, PDW, PCT, neutrophil, lymphocyte, albumin and SII values (*P* < *0.05*). Compared with the MDD group, the BD group exhibited higher MPV, PCT, neutrophil counts, lymphocyte counts, albumin levels and SII values (*P* < *0.05*), and lower platelet counts, RDW and PLR values (*P* < *0.05*). The data are shown in Table 2.

**Table 2:** Comparison of sociodemographic and laboratory variables among MDD, BD and healthy controls

Variables	MDD group (*n* = 8899)	BD group (*n* = 5108)	HC group (*n* = 6847)	F/^2^	*P*	*P1*	*P2*	*P3*	*P4*	*P5*	*P6*
Age (year)	44.95 ± 0.193^a^	39.19 ± 0.215^ab^	42.82 ± 0.157	211.320	0.000	0.000	0.000	0.000	0.000	0.000	0.000
Sex (male/female)	3193/5709^a^	2651/2457^ab^	2863/3984	342.416	0.000	0.000	0.000	0.000	0.000	0.000	0.000
Platelet	239.662 ± 0.665^a^	237.042 ± 0.894^ab^	252.994 ± 0.712	129.922	0.000	0.000	0.000	0.000	0.000	0.016	0.047
MPV	9.392 ± 0.011^a^	9.605 ± 0.016^ab^	8.699 ± 0.014	1201.846	0.000	0.000	0.000	0.000	0.000	0.000	0.000
PDW	13.764 ± 0.025^a^	13.859 ± 0.034^a^	15.931 ± 0.019	2276.150	0.000	0.000	0.000	0.000	0.000	0.042	0.126
PCT	0.208 ± 0.001^a^	0.211 ± 0.000^ab^	0.218 ± 0.001	74.552	0.000	0.000	0.000	0.000	0.000	0.000	0.001
Neutrophil	3.367 ± 0.015^a^	3.867 ± 0.024^ab^	3.597 ± 0.014	156.076	0.000	0.000	0.000	0.000	0.000	0.000	0.000
Lymphocyte	1.859 ± 0.006^a^	2.004 ± 0.009^b^	1.963 ± 0.007	52.257	0.000	0.000	0.000	0.764	1.000	0.000	0.000
RDW	13.691 ± 0.018^a^	13.501 ± 0.022^ab^	13.272 ± 0.014	144.082	0.000	0.000	0.000	0.000	0.000	0.000	0.000
Albumin	43.168 ± 0.038^a^	43.413 ± 0.056^ab^	45.999 ± 0.033	1629.851	0.000	0.000	0.000	0.000	0.000	0.000	0.000
PLR	140.599 ± 0.607^a^	130.039 ± 0.711^ab^	136.981 ± 0.545	20.193	0.000	0.000	0.000	0.000	0.000	0.000	0.000
SII	482.126 ± 3.945^a^	516.878 ± 5.656^ab^	492.205 ± 2.910	26.456	0.000	0.013	0.040	0.000	0.000	0.000	0.000
RPR	0.061 ± 0.000^a^	0.061 ± 0.000^a^	0.056 ± 0.000	150.147	0.000	0.000	0.000	0.000	0.000	0.713	1.000
PAR	5.578 ± 0.021	5.485 ± 0.021	5.520 ± 0.016	0.719	0.487	0.231	0.693	0.529	1.000	0.671	1.000

*HC* Healthy controls, *MDD* Major depressive disorder, *BD* Bipolar disorder, *MPV* Mean platelet volume, *PDW* Platelet distribution width, *PCT* Plateletcrit, *RDW* Red blood cell distribution width, *PLR* Platelet to lymphocyte ratio, *SII* Systemic immune-inflammation index, *RPR* RDW to platelet ratio, *PAR* Platelet to albumin ratio. a, vs. *HC* group, *P* < 0.05; b, vs. *MD*D, *P* < 0.05, *p* values after correction. *P*, comparison among *MDD* group, *BD* group and *HC* group; *P1*, *MDD group* vs *HC* group, *p* values uncorrected; *P2*, *MDD* group vs HC group, p values after correction; *P3*, BD group vs *HC* group, p values uncorrected; *P*4, *BD* group vs *HC* group, *p* values after correction; *P5*, *MDD* group vs *BD* group, p values uncorrected; *P6*, *MDD* group vs *BD* group, *p* values after correction. Values are represented as mean ± SEM

### Comparison of variables among first-episode MDD, recurrent MDD and healthy controls

The present study included 4,801 patients with first-episode MDD and 4,098 patients with recurrent MDD. The recurrent MDD group was older than the first-episode MDD group and the HC group, and the first-episode MDD group was younger than the HC group (*F* = 343.082, *P* < 0.001) in the present study. The Chi-square test rendered that the male/female ratio in the first-episode MDD group was lower than the HC group, the male/female ratio in the recurrent MDD group was lower than both the first-episode MDD group and the HC group (χ^2^ = 97.951, *P* < 0.001).

After adjusting for age and sex, the ANCOVA analysis demonstrated statistically significant differences in the platelet, MPV, PDW, PCT, neutrophil, lymphocyte, RDW, albumin, PLR, SII and RPR values among the three groups (*P* < *0.05*). Post hoc tests revealed that both the first-episode MDD group and the recurrent MDD possessed increased MPV, RDW, PLR and RPR values(*P* < *0.05*), and decreased platelet, PDW, PCT, neutrophil, lymphocyte and albumin levels (*P* < *0.05*) compared with the HC group. The first-episode MDD group had lower SII than HC group (*P* < *0.05*), while the recurrent MDD group had no difference in SII compared to th HC group (*P* > *0.05)*. Compared to the first-episode MDD group, the recurrent MDD group presented with higher neutrophil counts and lower albumin levels (*P* < *0.05*). The data are shown in Table 3.

**Table 3:** Comparison of sociodemographic and laboratory variables among first-episode MDD, recurrent MDD and healthy controls

Variables	First-episode MDD group (*n* = 4801)	Recurrent MDD group (*n* = 4098)	HC group (*n* = 6847)	F/^2^	*P*	*P1*	*P2*	*P3*	*P4*	*P5*	*P6*
Age (year)	41.10 ± 0.268^a^	49.46 ± 0.260^ab^	42.82 ± 0.157	343.082	0.000	0.000	0.000	0.000	0.000	0.000	0.000
Sex (male/female)	1868/2933^a^	1325/2773^ab^	2863/3984	97.951	0.000	0.002	0.006	0.000	0.000	0.000	0.000
Platelet	240.401 ± 0.907^a^	238.795 ± 0.978^a^	252.994 ± 0.712	99.641	0.000	0.000	0.000	0.000	0.000	0.530	1.000
MPV	9.398 ± 0.015^a^	9.386 ± 0.016^a^	8.699 ± 0.014	820.806	0.000	0.000	0.000	0.000	0.000	0.054	0.161
PDW	13.748 ± 0.034^a^	13.784 ± 0.037^a^	15.931 ± 0.019	2122.522	0.000	0.000	0.000	0.000	0.000	0.097	0.292
PCT	0.209 ± 0.001^a^	0.207 ± 0.001^a^	0.218 ± 0.001	75.593	0.000	0.000	0.000	0.000	0.000	0.089	0.266
Neutrophil	3.337 ± 0.020^a^	3.401 ± 0.022^ab^	3.597 ± 0.014	55.069	0.000	0.000	0.000	0.000	0.000	0.005	0.014
Lymphocyte	1.881 ± 0.009^a^	1.833 ± 0.009^a^	1.963 ± 0.007	43.691	0.000	0.000	0.000	0.000	0.000	0.079	0.236
RDW	13.696 ± 0.024^a^	13.684 ± 0.026^a^	13.272 ± 0.014	146.701	0.000	0.000	0.000	0.000	0.000	0.513	1.000
Albumin	43.313 ± 0.052^a^	42.999 ± 0.057^ab^	45.999 ± 0.033	1429.029	0.000	0.000	0.000	0.000	0.000	0.000	0.000
PLR	139.374 ± 0.820^a^	142.034 ± 0.901^a^	136.981 ± 0.545	3.279	0.038	0.015	0.045	0.000	0.000	0.017	0.050
SII	474.870 ± 5.490^a^	490.509 ± 5.657	492.205 ± 2.910	3.904	0.020	0.009	0.026	0.058	0.175	0.601	1.000
RPR	0.061 ± 0.000^a^	0.062 ± 0.000^a^	0.056 ± 0.000	116.035	0.000	0.000	0.000	0.000	0.000	0.379	1.000
PAR	5.579 ± 0.022	5.579 ± 0.024	5.520 ± 0.016	0.814	0.443	0.209	0.626	0.500	1.000	0.640	1.000

*HC* Healthy controls, *MDD* Major depressive disorder, *BD* Bipolar disorder, *MPV* Mean platelet volume, *PDW* Platelet distribution width, *PCT* Plateletcrit, *RDW* Red blood cell distribution width, *PLR*, Platelet to lymphocyte ratio, *SII* Systemic immune-inflammation index, *RPR* RDW to platelet ratio, *PAR* Platelet to albumin ratio, a, vs. HC group, *P* < 0.05; b, vs. First-episode *MDD*, *P* < 0.05, p values after correction. *P*, comparison among First-episode *MDD* group, Recurrent *MDD* group and *HC* group; *P1*, First-episode *MDD* group vs *HC* group, *p* values uncorrected; *P2*, First-episode *MDD* group vs *HC* group, *p* values after correction; *P3*, Recurrent *MDD* group vs *HC* group, *p* values uncorrected; *P*4, Recurrent *MDD* group vs *HC* group, *p* values after correction; *P5*, First-episode *MDD* group vs Recurrent *MDD* group, *p* values uncorrected; *P6*, First-episode *MDD* group vs Recurrent *MDD* group, p values after correction. Values are represented as mean ± SEM

### Comparison of variables among BD manic episodes, BD depressive episodes and healthy controls

A total of 3,444 patients with BD manic episodes and 1,664 patients with BD depressive episodes were recruited in the study. The comparison of age showed that both the BD manic episodes group and the BD depressive episodes group had lower age than the HC group (*P* < *0.05*), while there was no difference in age between the two BD groups (*P* > *0.05*). Comparison of sex showed that the male/female ratio was higher in the BD manic episodes group and the BD depressive episodes group than in the HC group (*P* < *0.05*), no difference was noted between the two BD groups (*P* > *0.05*).

The results displayed that the three groups had significant differences in platelet counts, MPV, PDW, PCT, neutrophil counts, RDW, albumin levels, PLR, SII, RPR and PAR values (*P* < 0.05). Further post hoc tests revealed that when compared to the HC group, both the BD manic episodes group and the BD depressive episodes group had increased MPV, RDW, and RPR values (*P* < 0.05), and decreased platelet counts, PDW, PCT, albumin and PLR values (*P* < 0.05); furthermore, the BD manic episodes group showed higher neutrophil counts and SII values than the HC group (*P* < 0.05) when BD depressive episodes group had no differences in neutrophil, SII with HC group (*P* > 0.05). Comparison with the BD depressive episodes group showed that the platelet counts, MPV, PCT, neutrophil counts, SII and PAR values were elevated (*P* < 0.05), and the albumin levels and RPR values were decreased (*P* < 0.05) in the BD manic episodes group. The data are shown in Table 4.

**Table 4:** Comparison of sociodemographic and laboratory variables among BD manic episodes, BD depressive episodes and healthy controls

Variables	BD depressive episodes group (n = 1664)	BD manic episodes group (n = 3444)	HC group (n = 6847)	F/^2^	*P*	*P1*	*P2*	*P3*	*P4*	*P5*	*P6*
Age(year)	39.35 ± 0.385^a^	39.11 ± 0.260^a^	42.82 ± 0.157	76.533	0.000	0.000	0.000	0.000	0.000	0.566	1.000
Sex (male/female)	836/828^a^	1815/1629^a^	2863/3984	122.471	0.000	0.000	0.000	0.000	0.000	0.099	0.297
Platelet	234.326 ± 1.544^a^	238.354 ± 1.096^ab^	252.994 ± 0.712	104.036	0.000	0.000	0.000	0.000	0.000	0.014	0.043
MPV	9.512 ± 0.027^a^	9.650 ± 0.019^ab^	8.699 ± 0.014	955.957	0.000	0.000	0.000	0.000	0.000	0.000	0.000
PDW	13.851 ± 0.058^a^	13.863 ± 0.041^a^	15.931 ± 0.019	1617.275	0.000	0.000	0.000	0.000	0.000	0.851	1.000
PCT	0.206 ± 0.001^a^	0.214 ± 0.001^ab^	0.218 ± 0.001	37.358	0.000	0.000	0.001	0.000	0.001	0.000	0.002
Neutrophil	3.670 ± 0.039	3.962 ± 0.031^ab^	3.597 ± 0.014	59.877	0.000	0.250	0.751	0.000	0.000	0.000	0.000
Lymphocyte	1.986 ± 0.016	2.012 ± 0.011	1.963 ± 0.007	0.865	0.421	0.590	1.000	0.311	0.934	0.225	0.676
RDW	13.557 ± 0.038^a^	13.474 ± 0.027^a^	13.272 ± 0.014	58.631	0.000	0.000	0.000	0.000	0.000	0.059	0.177
Albumin	43.642 ± 0.100^a^	43.303 ± 0.068^ab^	45.999 ± 0.033	1293.414	0.000	0.000	0.000	0.000	0.000	0.000	0.000
PLR	128.612 ± 1.269^a^	130.729 ± 0.965^a^	136.981 ± 0.545	13.316	0.038	0.000	0.000	0.001	0.002	0.075	0.226
SII	489.108 ± 9.022	530.295 ± 7.122^ab^	492.205 ± 2.910	21.994	0.000	0.859	1.000	0.000	0.000	0.000	0.000
RPR	0.062 ± 0.001^a^	0.061 ± 0.000^ab^	0.056 ± 0.000	99.604	0.000	0.000	0.000	0.000	0.000	0.011	0.033
PAR	5.394 ± 0.036	5.530 ± 0.026^b^	5.520 ± 0.016	6.756	0.001	0.019	0.058	0.035	0.105	0.000	0.000

*HC* Healthy controls, *BD* Bipolar disorder, *MPV* Mean platelet volume, *PDW* Platelet distribution width, *PCT* Plateletcrit, *RDW* Red blood cell distribution width, *PLR* Platelet to lymphocyte ratio, *SII* Systemic immune-inflammation index, *RPR* RDW to platelet ratio, *PAR* Platelet to albumin ratio; a, vs. *HC* group, *P* < 0.05; b, vs. *BD* depressive episodes, *P* < 0.05, p values after correction. *P*, comparison among *BD* depressive episodes group, *BD* manic episodes group and *HC* group; *P1*, *BD* depressive episodes group vs HC group, *p* values uncorrected; *P2*, *BD* depressive episodes group vs *HC* group, *p* values after correction; *P3*, *BD* manic episodes group vs *HC* group, *p* values uncorrected; *P*4, *BD* manic episodes group vs *HC* group, *p* values after correction; *P5*, *BD* depressive episodes group vs *BD* manic episodes group, *p* values uncorrected; *P6*, *BD* depressive episodes group vs *BD* manic episodes group, *p* values after correction. Values are represented as mean ± SEM

### MDD vs. BD depressive episodes and BD manic episodes

Comparison of age presented that age in the MDD group was higher than that in the BD depressive episodes group and the BD manic episodes group (*F* = 181.395, *P* < 0.001). The MDD group had a lower male/female ratio than the two BD groups (χ^2^ = 345.263, *P* < 0.001).

After adjusting for age and gender, ANCOVA revealed statistically significant differences in platelet counts, MPV, PCT, neutrophil counts, lymphocyte counts, RDW, albumin, PLR, SII, RPR and PAR values (*P* < 0.05) among the three groups. Post hoc tests showed that after adjusting for age and sex, the MDD group possessed higher platelet counts, RDW, PLR and PAR values (*P* < 0.05), and lower MPV, neutrophil counts and lymphocyte counts (*P* < 0.05) than the BD depressive episodes group. When compared to the BD manic episodes group, the RDW and PLR values in the MDD group were increased (*P* < 0.05), the MPV, PCT, neutrophil counts, lymphocyte counts, albumin and SII values in MDD group were decreased (*P* < 0.05). The BD manic group had the highest MPV, PCT, neutrophil counts and SII values of the three groups, and the MDD group had the highest RDW and PLR of the three groups. The data are shown in Table 5.

**Table 5:** Comparison of sociodemographic and laboratory variables between the MDD group with the BD manic episodes group or with the BD depressive episodes group

Variables	MDD group (*n* = 8899)	BD depressive episodes group (*n* = 1664)	BD manic episodes group (*n* = 3444)	t/F/^2^	*P*	*P1*	*P2*	*P3*	*P4*	*P5*	*P6*
Age(year)	44.95 ± 0.193^ab^	39.35 ± 0.385	39.11 ± 0.260	181.395	0.000	0.000	0.000	0.000	0.000	0.640	1.000
Sex (male/female)	3193/5709^ab^	836/828	1815/1629	345.263	0.000	0.000	0.000	0.000	0.000	0.099	0.297
Platelet	239.662 ± 0.665^a^	234.326 ± 1.544	238.354 ± 1.096^a^	4.260	0.014	0.004	0.011	0.764	1.000	0.016	0.048
MPV	9.392 ± 0.011^ab^	9.512 ± 0.027	9.650 ± 0.019^a^	57.513	0.000	0.001	0.003	0.000	0.000	0.000	0.000
PDW	13.764 ± 0.025	13.851 ± 0.058	13.863 ± 0.041	1.975	0.139	0.237	0.711	0.068	0.204	0.854	1.000
PCT	0.208 ± 0.001^b^	0.206 ± 0.001	0.214 ± 0.001^a^	17.759	0.000	0.196	0.589	0.000	0.000	0.000	0.000
Neutrophil	3.367 ± 0.015^ab^	3.670 ± 0.039	3.962 ± 0.031^a^	145.385	0.000	0.000	0.000	0.000	0.000	0.000	0.000
Lymphocyte	1.859 ± 0.006^ab^	1.986 ± 0.016	2.012 ± 0.011	31.449	0.000	0.000	0.000	0.000	0.000	0.226	0.678
RDW	13.691 ± 0.018^ab^	13.557 ± 0.038	13.474 ± 0.027	15.521	0.000	0.016	0.048	0.000	0.000	0.226	0.678
Albumin	43.168 ± 0.038^b^	43.642 ± 0.100	43.303 ± 0.068^a^	16.010	0.000	0.787	1.000	0.000	0.000	0.000	0.001
PLR	140.599 ± 0.607^ab^	128.612 ± 1.269	130.729 ± 0.965	17.704	0.000	0.000	0.000	0.000	0.000	0.115	0.346
SII	482.126 ± 3.945^b^	489.108 ± 9.022	530.295 ± 7.122^a^	26.774	0.000	0.132	0.397	0.000	0.000	0.000	0.000
RPR	0.061 ± 0.000	0.062 ± 0.001	0.061 ± 0.000^a^	3.874	0.021	0.032	0.096	0.215	0.646	0.005	0.016
PAR	5.578 ± 0.021^a^	5.394 ± 0.036	5.530 ± 0.026^a^	5.892	0.000	0.006	0.018	0.169	0.507	0.001	0.002

*HC* Healthy controls, *MDD* Major depressive disorder, *BD* Bipolar disorder, *MPV* Mean platelet volume, *PDW* Platelet distribution width, *PCT* Plateletcrit, *RDW* Red blood cell distribution width, *PLR* Platelet to lymphocyte ratio, *SII* Systemic immune-inflammation index, *RPR* RDW to platelet ratio, *PAR* Platelet to albumin ratio; a, vs. *BD* depressive episodes group, *P* < 0.05; b, vs. *BD* manic episodes, *P* < 0.05, *p* values after correction. *P*, comparison among *MDD* group, *BD* depressive episodes group and *BD* manic episodes group; *P1*, *MDD* group vs *BD* depressive episodes group, *p* values uncorrected; *P2*, *MDD* group vs *BD* depressive episodes group, *p* values after correction; *P3*, *MDD* group vs *BD* manic episodes group, *p* values uncorrected; *P4*, *MDD* group vs *BD* manic episodes group, *p* values after correction; *P5*, *BD* depressive episodes group vs *BD* manic episodes group, *p* values uncorrected; *P6*, *BD* depressive episodes group vs *BD* manic episodes group, *p* values after correction. Values are represented as mean ± SEM

### Comparison of haematological parameters between males and females

**Fig. 1:**
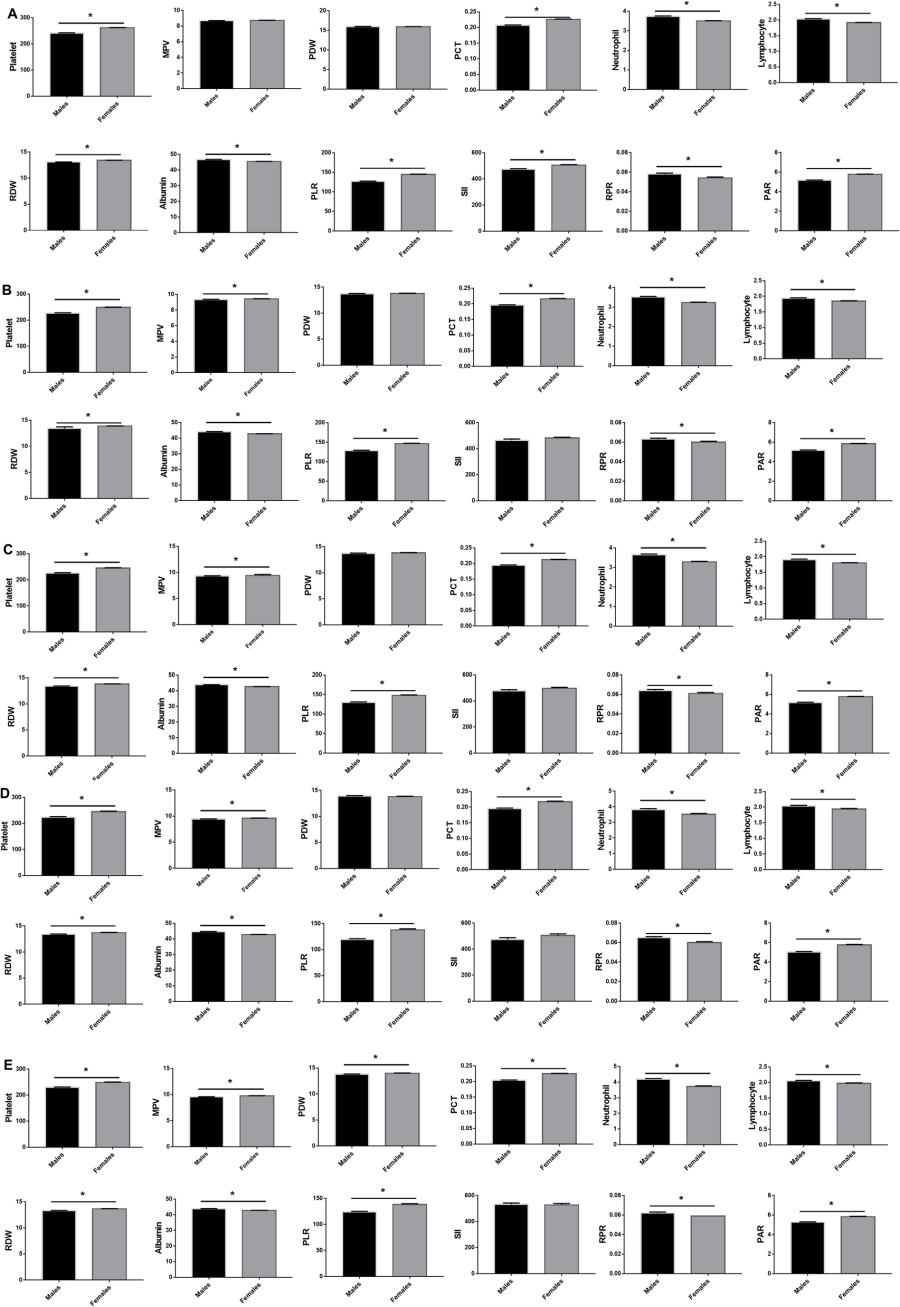
Comparison of haematological parameters between males and females in HC group **A**, first-episode MDD group **B**, recurrent MDD group **C**, BD depressive episodes group **D** and BD manic episodes group **E**. Data are represented as the mean ± SEM; *, vs. Female group, *P* < 0.05; HC, healthy controls; MDD, major depressive disorder; BD, bipolar disorder

We also analysed differences in haematological parameters between males and females. In the HC group, there were 2,863 males and 3,984 females, the males presented higher neutrophil counts, lymphocyte counts, albumin and RPR values (*P* < 0.05), and lower platelet counts, PCT, RDW, PLR, SII and PAR values (*P* < 0.05) than females. The data are shown in Fig. 1A.

There were 1,868 males and 2,933 females in the first-episode MDD group. The males showed higher neutrophil, lymphocyte, albumin and RPR values (*P* < 0.05), and lower platelet, MPV, PCT, RDW, PLR and PAR values (*P* < 0.05) than the females. The data are shown in Fig. 1B.

The recurrent MDD group included 1,325 males and 2,773 females. Comparison of blood test parameters between males and females in the recurrent MDD group revealed that the neutrophil, lymphocyte, albumin and RPR values were increased (*P* < 0.05), and the platelet, MPV, PCT, RDW, PLR and PAR values were decreased (*P* < 0.05) in males compared to those in females The data are shown in Fig. 1C.

In the BD depressive episodes group, 836 males and 823 females were included. The comparison showed that the neutrophil, lymphocyte, albumin and RPR values in males were higher than those in females (*P* < 0.05), The values of platelet, MPV, PCT, RDW, PLR and PAR values in males were lower than those in females (*P* < 0.05). The data are shown in Fig. 1D.

There were 1,815 males and 1,629 females in the BD manic episodes group. When compared to the females, the males in the BD manic episodes group presented higher neutrophil, lymphocyte, albumin and RPR values (*P* < 0.05), and lower platelet, MPV, PDW, PCT, RDW, PLR and PAR values (*P* < 0.05). The data are shown in Fig. 1E.

### Correlations between haematological parameters and age

Spearman correlation analysis was used to test the correlations between the haematological parameters and age in the HC group and the diagnostic groups. In the HC group, the PDW, neutrophil, RDW, SII and RPR values were positively correlated with age (*P* < 0.05), the platelet, MPV, PCT, lymphocyte, albumin and PAR values were negatively correlated with age (*P* < 0.05).

In the first-episode MDD group, the Spearman correlation analysis showed that there were positive relationships between PLR, SII, RPR values and age (*P* < 0.05), there were negative relationships between platelet, MPV, PDW, PCT, lymphocyte, albumin values and age (*P* < 0.05).

In the recurrent MDD group, the PLR, SII and RPR values were positively correlated with age (*P* < 0.05), platelet, MPV, PDW, PCT, neutrophil, lymphocyte, albumin, and PAR values were negatively correlated with age (*P* < 0.05).

In the BD depressive episodes group, the Spearman correlation analysis revealed that the PLR, SII and RPR had positive correlations with age (*P* < 0.05), but platelet, MPV, PCT, lymphocyte and albumin values had negative correlations with age (*P* < 0.05).

In the BD manic episodes group, the PLR and RPR values were positively correlated with age (*P* < 0.05), and the platelet, MPV, PCT, neutrophil, lymphocyte and albumin values were negatively correlated with age (*P* < 0.05). The data are shown in Table 6.

**Table 6:** Correlations between hematological parameters and age in diagnostic groups

Parameters	Groups				
	HC	First-episode MDD	Recurrent MDD	BD depressive episodes	BD manic episodes
Platelet	-0.111**	-0.115**	-0.131**	-0.114**	-0.126**
MPV	-0.043**	-0.185**	-0.134**	-0.142**	-0.039*
PDW	0.061**	-0.042**	-0.037*	-0.021	-0.005
PCT	-0.135**	-0.214**	-0.207**	-0.177**	-0.162**
Neutrophil	0.035**	0.003	-0.048**	-0.007	-0.050**
Lymphocyte	-0.109**	-0.310**	-0.214**	-0.230**	-0.203**
RDW	0.110**	0.025	-0.006	-0.008	-0.024
Albumin	-0.275**	-0.294**	-0.268**	-0.221**	-0.278**
PLR	0.014	0.196**	0.097**	0.115**	0.092**
SII	0.030*	0.132**	0.033*	0.070**	0.025
RPR	0.129**	0.086**	0.108**	0.109**	0109**
PAR	-0.038*	-0.017	-0.041**	-0.030	-0.031

Results given as Spearman's correlation coefficient. *HC* Healthy controls, *MDD* Major depressive disorder, *BD* Bipolar disorder, *MPV* Mean platelet volume, *PDW* Platelet distribution width, *PCT* Plateletcrit, *RDW* Red blood cell distribution width, *PLR* Platelet to lymphocyte ratio, *SII* Systemic immune-inflammation index, *RPR* RDW to platelet ratio, *PAR* Platelet to albumin ratio; *, *P* < 0.05; **, *P* < 0.01

### The haematological parameter predictors of the diagnosis of MDD or BD

**Fig. 2:**
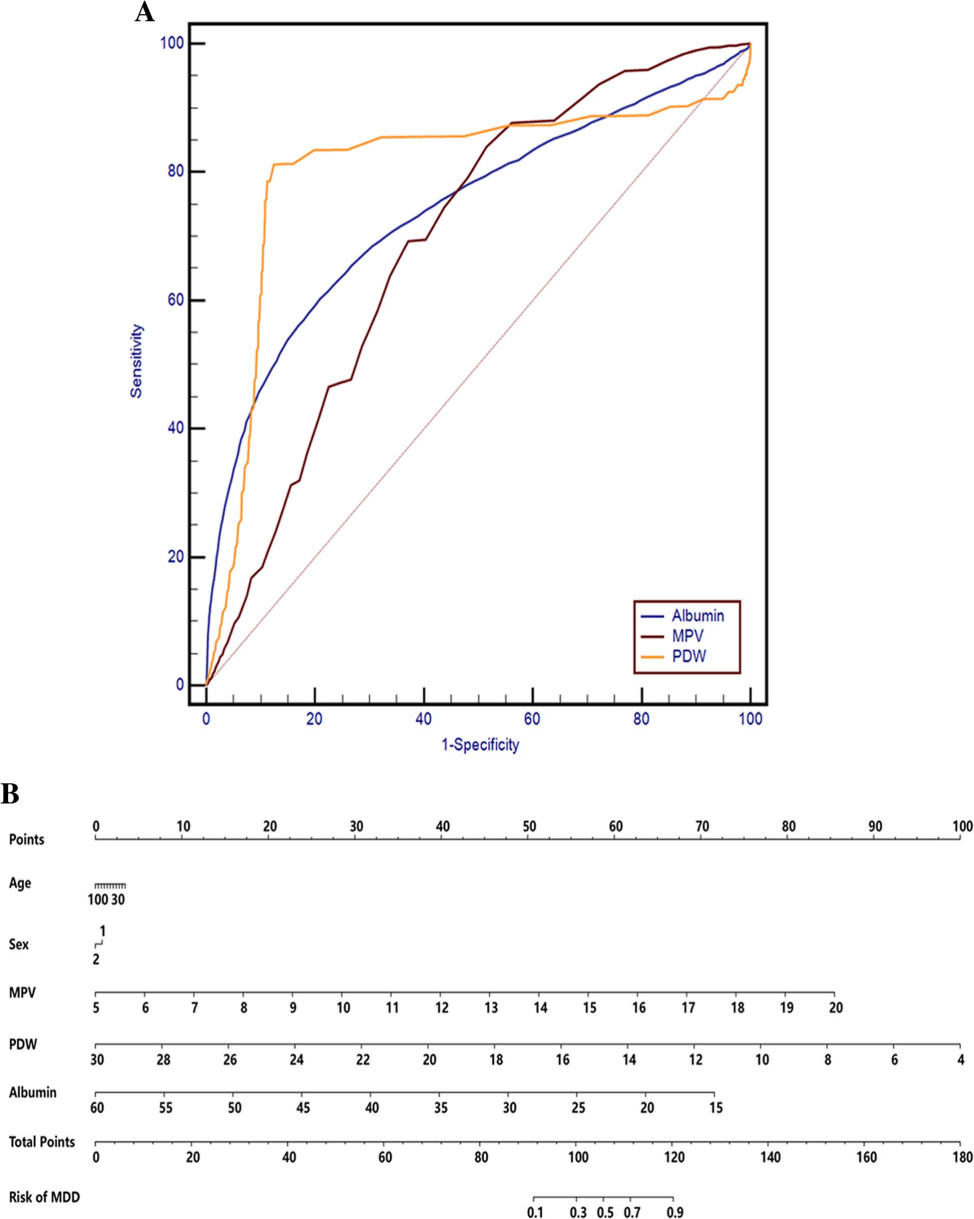
**A** ROC curves for the diagnostic ability of MPV, PDW and Albumin for MDD (MDD vs. healthy controls). PDW: AUC 0.804 (95% CI = 0.791 to 0.810), *P* < 0.001; Albumin: AUC 0.742 (95% CI = 0.735 to 0.749), *P* < 0.001; MPV: AUC 0.693 (95% CI = 0.686 to 0.701), *P* < 0.001. **B** Risk factors of MDD nomogram. (Code of sex, 1: male, 2: female) (To use the nomogram, an individual patient’s value is located on each variable axis, and a line is drawn upward to determine the number of points received for each variable value. The sum of these numbers is located on the Total Points axis, and a line is drawn downward to the Risk of MDD axes to determine the MDD risk). MDD: major depressive disorder; AUC: area under the ROC curve, MPV: mean platelet volume; PDW, platelet distribution width

Based on the ROC curve analysis for the diagnosis of MDD, the parameters with an area under curve (AUC) higher than 0.7 were the PDW and albumin, the AUC of MPV was close to 0.7, the AUC of other parameters were lower than 0.6. The PDW cut-off point of 15.5% which showed 81.16% sensitivity and 87.62% specificity, and with the AUC 0.804 (95% CI: 0.791–0.810) was the optimal cut-off point for the identification of MDD. Albumin cut-off point of 43.8 g/L which displayed 60.24% sensitivity and 79.11% specificity, and with the AUC 0.742 (95% CI: 0.735–0.749) was the optimal cut-off point for identification of MDD. Furthermore, 8.4 fL as a cut-off value for MPV differentiated patients with MDD from healthy controls with a sensitivity of 83.91% and a specificity of 48.55% (AUC: 0.693, 95%CI: 0.686–0.701, *P* < 0.001). The data are shown in Fig. 2A.

In the binary logistic regression analysis in MDD, the disease state was used as the dependent variable, and age, sex, MPV, PDW and albumin were used as covariates, the forward conditional method was employed. Collinearity diagnostics showed that the largest value of variance inflation factor (VIF) was 1.255, suggesting that there was no obvious collinearity between the variables. The results showed that age, sex, MPV, PDW and albumin (*P* < 0.001) were independent indicators for MDD (Table 7). Nomogram was applied to predict the risk of MDD using a combination of multiple indicators. The data are shown in Fig. 2B.

**Table 7:** Independent influence factors of MDD or BD by binary logistic regression analysis

Variables	MDD				BD			
	β	*P*	OR	*95%CI*	β	*P*	OR	*95%CI*
Age	-0.005	0.000	0.995	0.992—0.998	-0.038	0.000	0.962	0.959—0.966
Sex	0.119	0.008	1.126	1.032—1.229	0.819	0.000	2.268	2.054—2.506
MPV	0.861	0.000	2.366	2.265—2.472	0.717	0.000	2.048	1.960—2.139
PDW	-0.581	0.000	0.559	0.547—0.571	-0.495	0.000	0.610	0.596—0.624
Albumin	-0.240	0.000	0.786	0.775—0.789	-0.251	0.000	0.778	0.765—0.791

*MPV* Mean platelet volume, *PDW* Platelet distribution width

**Fig. 3:**
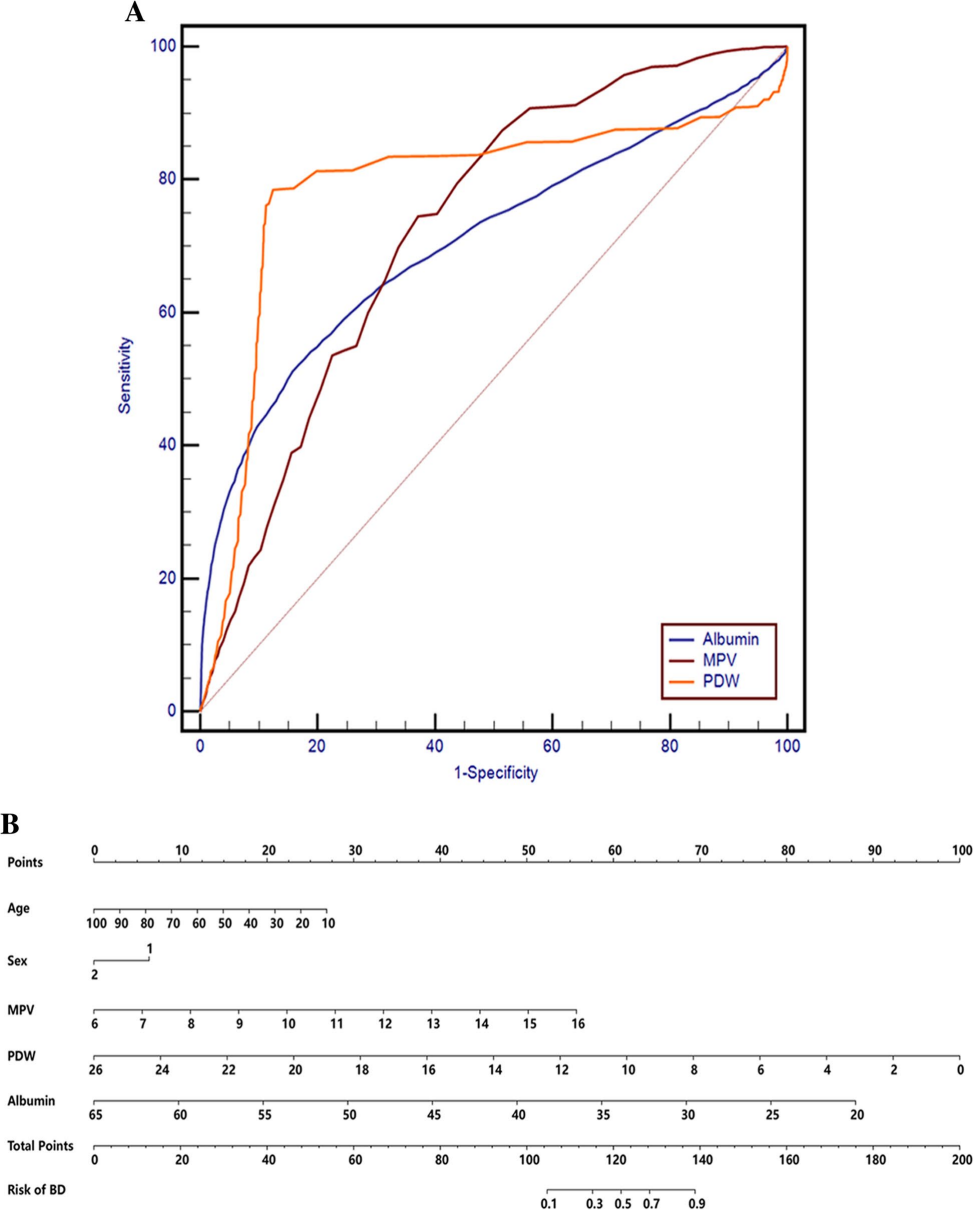
**A** ROC for the diagnostic ability of MPV, PDW and Albumin for BD (BD vs. healthy controls). ROC curves for MPV, PDW and Albumin values for the diagnosis of BD. PDW: AUC 0.789 (95% CI = 0.781 to 0.796), *P* < 0.001; MPV: AUC 0.731 (95% CI = 0.723 to 0.739), *P* < 0.001; Albumin: AUC 0.708 (95% CI = 0.699 to 0.716), *P* < 0.001. (**B**) Risk factors of BD nomogram. (Code of sex, 1: male, 2: female) (To use the nomogram, an individual patient’s value is located on each variable axis, and a line is drawn upward to determine the number of points received for each variable value. The sum of these numbers is located on the Total Points axis, and a line is drawn downward to the Risk of BD axes to determine the BD risk). BD: bipolar disorder; AUC: area under the ROC curve, MPV: mean platelet volume; PDW, platelet distribution width

The ROC curve analysis for the diagnosis of BD (Fig. 3A) showed that the parameters with AUCs higher than 0.7 were MPV, PDW and albumin, the AUCs of other variables were lower than 0.6. A cut-off value of 15.5% for PDW (AUC: 0.789, 95% CI: 0.781–0.0.796, P < 0.001) differentiated patients with BD from healthy controls with a sensitivity of 78.45% and a specificity of 87.62%. An MPV cut-off point of 8.8 fL (AUC: 0.731, 95% CI: 0.723–0.739) which showed 74.45% sensitivity and 62.92% specificity was the optimal cut-off point to differentiate patients with BD from healthy controls. Furthermore, an albumin cut-off point of 43.3 g/L which displayed 51.17% sensitivity and 84.26% specificity, and with the AUC 0.708 (95% CI: 0.699–0.716) was the optimal cut-off point for the identification of BD.

The binary logistic regression analysis in BD, with disease state as the dependent variable, and age, sex, MPV, PDW and albumin as covariates, used the forward conditional method. Collinearity diagnostics showed that the largest value of the variance inflation factor (VIF) was 1.205, suggesting that there was no obvious collinearity. The results demonstrated that the age, sex, MPV, PDW and albumin (*P* < 0.001) were independent contributing factors for the occurrence of BD (Table 7). Nomogram was applied to predict the risk of BD using a combination of multiple indicators. The data are shown in Fig. 3B.

## Discussion

To the best of our knowledge, the present study was the first large-scale study to assess the platelet parameters, including platelet counts, MPV, PDW, PCT, PLR, SII, RPR and PAR, in individuals with first-episode MDD, recurrent MDD, BD depressive episodes and BD manic episodes. These observations are new, since no previously published studies have compared these platelet parameters across these clinical groups.

The present study revealed that, in the subjects, the patients with BD manic episodes had the highest mean values of MPV and SII, the patients with BD depressive episodes had the lowest mean values of platelet counts and PAR, and the patients with MDD group had the highest mean values of PLR and RDW. The present study found that both patients with first-episode MDD and recurrent MDD displayed higher MPV, PLR and RPR values and lower platelet counts, PDW and PCT values than healthy controls. Meanwhile, compared to healthy controls, both patients with BD manic episodes and BD depressive episodes had increased MPV and RPR values, and decreased platelet counts, PDW, PCT and PLR values; patients with BD manic episodes showed higher platelet counts, MPV, PCT, SII and PAR values and lower RPR values than patients with BD depressive episodes. Compared with the MDD patients, the MPV, PCT and SII values in BD patients were increased, especially in the patients with manic episodes; the platelet counts, and PLR were decreased. The observation also showed that some platelet parameters had differences between the males and females, and had correlations with age. Our results also indicated that high MPV levels, low PDW levels and low albumin levels were independent predictors for differentiating patients with MDD or BD from healthy controls.

Platelets are increasingly considered to play an important role in inflammation, acting as a first-line inflammatory response that regulates the permeability of endothelial cells and the recruitment of neutrophils, macrophages and their effectors [8]. At present, platelets are being condidered as a putative bridge linking mental diseases and inflammatory response [11]. Chronic inflammation usually includes a lower grade inflammatory response which is a weakened but persistent form of the inflammatory response. Chronic inflammation plays a role in the pathophysiology of many psychiatric diseases including affective disorders and schizophrenia [37]. Chronic inflammation may impair mood, cognition, motivation, appetite and sleep in patients with affective disorders [38, 39]. One hypothesis is that inflammatory substances reveal the destabilization of the brain function, effected with environmental factors and, accordingly contribute to the presentation of mood symptoms [40]. In the present study, we found that the patients with MDD and BD had lower platelet counts than the HC group, and the platelet counts in BD patients were lower than those in MDD patients. The patients with BD manic episodes had higher platelet levels than patients with BD depressive episodes, and the patients with BD depressive episodes had the lowest platelet counts. Our results concur with one previous study which found that patients with unipolar depression had higher platelet levels than BD patients [41]. Other studies found that in BD patients, those experiencing (hypo)manic episodes had higher platelet levels than those experiencing depressive episodes or euthymic states [42, 43]. Inconsistent results also exist, some studies have not found differences in the platelet counts of MDD and BD patients [44, 45].

MPV is closely correlated with platelet reactivity, previous studies have reported that in some inflammatory diseases, the levels of MPV are increased and can serve as a potential inflammatory marker [8]. Our study showed that both patients with MDD and BD had elevated MPV levels, the patients with first-episode and recurrent MDD both had elevated MPV values, MPV levels in the BD manic episodes group were higher than those in the BD depressive episodes group and the MDD group, and the patients with BD manic episodes had the highest MPV. These results suggest a severe dysregulation of platelet activity in BD mania episodes. Previous studies found that the MPV in MDD patients was significantly higher than that in healthy controls, and 8 weeks of treatment with escitalopram retured MPV to normal levels [46]. A study of BD patients showed that BD patients had higher MPV than controls [47]. The results were consistent with our findings. In our study, the logistic analysis and ROC analysis showed that high MPV levels were independently correlated with MDD or BD, suggesting that it can be a an independent predictor differentiating patients with MDD or BD from healthy controls.

PDW and PCT are markers reflecting platelet activation. The present study showed that the PDW and PCT levels in patients with MDD and BD were significantly lower than those in healthy contols. The patients with MDD and BD depressive episodes had lower PCT levels than patients with BD manic episodes, the MDD group had no difference in PCT with BD depressive episodes grooup, and no differences in PDW among the three groups were found. RDW is an indicator of red blood cell size, in recent years, it has been used as an inflammatory marker by scholars to study its relationship with inflammation. Our study found that the patients with MDD and BD both had increased RDW levels compared to HCs, the MDD patients had higher RDW than BD patients, no differences in RDW were found between patients with BD depressive episodes and BD manic episodes. Regarding PDW, PCT and RDW, inconsistent results has been reported in psychiatic disorders. In a small study, Aleksovski et al. compared 31 patients with lifelong recurrent depression treated with selective serotonin reuptake inhibitors with 31 healthy controls and found significant increases in MPV and PDW in depression patients [48]. A study involving 103 patients with MDD and 106 healthy controls showed that the MDD groups had significantly higher PCT, PLR, RDW and MPV than the controls, and no differences in PDW between the two groups were shown [49]. In a study of patients with first-episode schizophrenia (FES) and first-episode depression (FED), the results showed that the levels of MPV, PDW and PCT in FES patients were higher than those in healthy controls (HCs), the PCT values in FED were higher than those in the HCs, no significant difference in MPV or PDW was identified between FED patients and HCs [50]. A study of patients with BD found that the median MPV and PCT values of patients with BD were significantly greater than those of the control group, and they did not find alteration in RDW in BD patients [47]. The reason for the inconsistency may lie in the differences in sample size and study populations. The results of the present large-scale study showed that low PDW levels were independently related to MDD or BD, suggesting that it may be considered a biomarker differentiating Chinese patients who have MDD or BD from healthy population.

PLR, SII, RPR and PAR are the inflammation ratios initially used to assess the prognosis of patients with physical health disorders, including cancer and coronary heart disease, are now thought to reflect inflammatory status [51, 52]. Our results showed that in patients with MDD, the PLR and RPR levels were elevated, and SII levels were decreased. In patients with BD, the PLR levels were decreased, and the RPR and SII levels were increased. The patients with BD manic episodes had the highest mean SII values, the MDD group had the highest mean PLR values, the patients with BD depresisive episodes had the lowest mean PAR values. Few studies have tested these inflammation ratios in individuals with affective disorders, and the results are inconsistent. One study showed that PLRs in patients experiencing BD (Hypo)manic episodes were higher than in those experiencing depressive episodes, a meta-analysis showed that PLRs in BD patients were higher than that in control subjects [53], PLR has been recently suggested as a marker of high-lethality suicide attempts [54], while other studies reported no alterations of PLRs in MDD and BD patients [44, 45]. One previous study on depression in adolescent girls showed that PLR and RPR values were significantly higher in subjects with a high depression score than in normal individuals [33], which is consistent with our results in MDD. Jie Wang et al. studied the association between SII and diabetic depression, and found that the SII levels in diabetic mellitus patients with depression were higher than those in diabetic mellitus patients without depression [29], and their mean SII values were all higher than our data in the present study. The inconsistency of results may be due to differences in the population studied and sample sizes of these studies.

The present study also found that patients with BD had increased neutrophil counts compared with HCs, and had increased neutrophil and lymphocyte levels compared with MDD patients. Patients in the BD manic episodes group showed higher neutrophil counts than patients with BD depressive episodes and the HCs. Patients with BD depressive episodes had no differences in neutrophil counts compared to the HCs, and had no differences in lymphocyte levels compared to patients with BD manic episodes and the HCs. Patients with first-episode MDD and recurrent MDD both showed lower neutrophil and lymphocyte counts than HCs. Neutrophils are the first line of cellular defence against infection as a key cell type in the innate immune system, and have phagocytic and apoptotic functions, lymphocytes are involved in the adaptive immune response and are specific inflammatory mediators that present a regulatory or protective effect [45]. Immune responses to various injuries are often characterized by an increase in neutrophils and a decrease in lymphocyte counts, and proinflammatory cytokines may lead to an increase in the absolute count of neutrophils and a decrease in the absolute count of lymphocytes [55]. Previous studies showed that neutrophils were significantly higher in patients experiencing a manic episode than in depressed individuals, and the lymphocyte levels did not differ between the two groups [42], which is consistent with the results of our study. A previous study of BD patients showed that the median neutrophil and lymphocyte values in BD patients were higher than that in control group [47]. A small sample study showed that the patients with MDD had significantly greater neutrophil counts and lower lymphocyte counts than HCs [49]. These results, as well as our findings, suggest that MDD and BD have a dysregulation of the inflammatory system.

Albumin, a non-enzymatic antioxidants, is the reactant in the acute phase of the systemic inflammatory response, and it is a negative acute phase protein whose serum level is downregulated in the inflammatory state [56, 57]. Decreased albumin levels have been shown in MDD and BD patients in comparison with healthy controls. A meta analysis involving 115 articles showed that albumin levels in patients with MDD were lower than that in controls, and were increased after antidepressant therapy [58]. A study of patients with BD showed that patients in the manic state and depressive state had lower albumin levels than patients in the euthymic state and healthy controls [59]. One previous study comparing albumin levels among patients with MDD, bipolar mania and bipolar depression showed no differences among the three groups [60]. Our study showed that after adjusting for age and sex, patients with MDD and BD both had decreased albumin levels compared with HCs, and the differences in albumin levels among patients with MDD, BD mania and BD depression were significant. Further analysis revealed that albumin levels in both patients with first-episode and recurrent MDD were decreased, and the decline was more obvious in the patients with recurrent MDD. BD patients experiencing manic episodes and depressive episodes both showed decreased albumin levels compared with HCs, and the decline in former was more obvious. The results also showed that the albumin levels in females were lower than those in males, and the albumin levels were negatively correlated with age, suggesting that it is necessary to adjust for sex and age when comparing the differences between groups. In the present study, logistic regression showed the albumin was an independent influence factor for MDD or BD, ROC analysis suggested that albumin was significantly predictive in differentiating patients with MDD or BD from healthy controls.

There are some strengths and limitations in the present study. First, the study analysed the parameters of 14,007 patients with affective disorders and 6,847 healthy controls. To the best of our knowledge, this is the largest cross-sectional study that explored the alteration of peripheral platelet parameters in patients experiencing first-episode MDD, recurrent MDD, BD manic episodes and BD depressive episodes. Furthermore, the real-world measures in the study increased the clinical transferability of our results. Meanwhile, the study compared the platelet parameters between first-episode MDD and recurrent MDD, to our knowledge, this is the first study to evaluate these parameters in these particular populations. Nevertheless, some limitations should be discussed. For instance, the retrospective design of the study did not allow us to conduct a structured psychiatric interview, and the severity of symptoms was not analysed in the present study. Second, the study did not take into account the effects of treatment, which may affect the levels of the parameters. Third, the cross-sectional design apparently did not allow causal inferences about the direction of the relationship between platelet parameters and the onset of different affective disorders. Longitudinal studies are needed to investigate the interaction between platelet parameters and different affective disorders over time. Fourth, some confounding factors may influence the levels of platelet parameters, and we were not able to control for some factors in the present study, such as smoking, diet, and body mass index.

## Conclusions

In conclusion, the present study demonstrated that platelet parameters were altered in patients with affective disorders, and there were significant differences in platelet parameters among patients with first-episode MDD, recurrent MDD, BD manic episodes and BD depressive episodes. The patients with BD manic episodes had the highest mean values of MPV and SII, the patients with BD depressive episodes had the lowest mean values of platelet counts and PAR, and the patients with MDD had the highest mean values of PLR and RDW, suggesting that different affective disorders have unique platelet parameter variation patterns. Patients with MDD displayed increased MPV, PLR, RPR and RDW values, and decreased paltelet, neutrophil, lymphocyte, albumin, PDW, PCT and SII values; patients with BD showed increased MPV, neutrophil, RDW, RPR and SII values, and decreased platelet, PDW, PCT, albumin and PLR values. The MPV, PCT and SII values in patients with BD were increased compared with that in patients with MDD, the increases were more obvious in the patients with manic episodes, and the platelet and PLR values in BD patients were decreased compared with that in MDD patients. The observation also showed that some platelet parameters had differences between sexes, and had correlations with age. Our results also indicated that MPV, PDW and albumin levels were independently correlated with MDD and BD, and ROC curve analysis showed that MPV, PDW and albumin levels are important predictors for differentiating patients with MDD or BD from healthy controls, they may be peripheral trait biomarkers, which may reflect enhanced inflammatory signalling in affective disorders. Our study demonstrated the role of platelet parameters and systemic inflammation in the pathophysiology of MDD and BD. As platelet parameters are simple and inexpensive examinations that are routinely obtained in inpatients and outpatients, further longitudinal studies may discover more solid links between platelet parameters and affective disorders.

## Data Availability

The datasets used and/or analyzed during the current study are available from the corresponding author on reasonable request.

## References

[1] Rehm J, Shield KD (2019). Global Burden of Disease and the Impact of Mental and Addictive Disorders. Curr Psychiatry Rep.

[2] Jones B, Daskalakis ZJ, Carvalho AF, Strawbridge R, Young AH, Mulsant BH, Husain MI (2020). Inflammation as a treatment target in mood disorders: review. BJPsych Open.

[3] Grande I, Berk M, Birmaher B, Vieta E (2016). Bipolar disorder. Lancet.

[4] Kupfer DJ, Frank E, Phillips ML (2016). Major depressive disorder: new clinical, neurobiological, and treatment perspectives. Focus (Am Psychiatr Publ).

[5] Rhie SJ, Jung EY, Shim I (2020). The role of neuroinflammation on pathogenesis of affective disorders. J Exerc Rehabil.

[6] Voineskos D, Daskalakis ZJ, Blumberger DM (2020). Management of treatment-resistant depression: challenges and strategies. Neuropsychiatr Dis Treat.

[7] Rush AJ, Trivedi MH, Wisniewski SR, Nierenberg AA, Stewart JW, Warden D, Niederehe G, Thase ME, Lavori PW, Lebowitz BD (2006). Acute and longer-term outcomes in depressed outpatients requiring one or several treatment steps: a STAR*D report. Am J Psychiatry.

[8] Pogorzelska K, Kretowska A, Krawczuk-Rybak M, Sawicka-Zukowska M (2020). Characteristics of platelet indices and their prognostic significance in selected medical condition - a systematic review. Adv Med Sci.

[9] Canobbio I (2019). Blood platelets: circulating mirrors of neurons?. Res Pract Thromb Haemost.

[10] Goubau C, Buyse GM, Di Michele M, Van Geet C, Freson K (2013). Regulated granule trafficking in platelets and neurons: a common molecular machinery. Eur J Paediatr Neurol.

[11] Izzi B, Tirozzi A, Cerletti C, Donati MB, de Gaetano G, Hoylaerts MF, Iacoviello L, Gialluisi A (2020). Beyond haemostasis and thrombosis: platelets in depression and its co-morbidities. INT J MOL SCI.

[12] Koupenova M, Clancy L, Corkrey HA, Freedman JE (2018). Circulating platelets as mediators of immunity, inflammation, and thrombosis. CIRC RES.

[13] Salamanna F, Maglio M, Landini MP, Fini M (2020). Platelet functions and activities as potential hematologic parameters related to Coronavirus Disease 2019 (Covid-19). Platelets.

[14] Bakogiannis C, Sachse M, Stamatelopoulos K, Stellos K (2019). Platelet-derived chemokines in inflammation and atherosclerosis. Cytokine.

[15] Budak YU, Polat M, Huysal K (2016). The use of platelet indices, plateletcrit, mean platelet volume and platelet distribution width in emergency non-traumatic abdominal surgery: a systematic review. Biochem Med (Zagreb).

[16] Osselaer JC, Jamart J, Scheiff JM (1997). Platelet distribution width for differential diagnosis of thrombocytosis. CLIN CHEM.

[17] Wang Z, Liu C, Fang H (2019). Blood Cell Parameters and Predicting Coronary In-Stent Restenosis. Angiology.

[18] Atak BM, Duman TT, Aktas G, Kocak MZ, Savli H (2018). Platelet distribution width is associated with type 2 diabetes mellitus and diabetic nephropathy and neuropathy. National Journal of Health Sciences.

[19] Aktas G, Duman T, Atak B, Kurtkulagi O, Bilgin S, Basaran E, Demirkol M, Kosekli M (2020). Irritable bowel syndrome is associated with novel inflammatory markers derived from hemogram parameters. Family Medicine & Primary Care Review.

[20] Sincer I, Mansiroglu AK, Erdal E, Cosgun M, Aktas G, Gunes Y (2020). Could platelet distribution width predict coronary collateral development in stable coronary artery disease?. North Clin Istanb.

[21] Demirin H, Ozhan H, Ucgun T, Celer A, Bulur S, Cil H, Gunes C, Yildirim HA (2011). Normal range of mean platelet volume in healthy subjects: Insight from a large epidemiologic study. THROMB RES.

[22] Giles H, Smith RE, Martin JF (1994). Platelet glycoprotein IIb-IIIa and size are increased in acute myocardial infarction. EUR J CLIN INVEST.

[23] Aktas G, Sit M, Tekce H, Alcelik A, Savli H, Simsek T, Ozmen E, Isci AZ, Apuhan T (2013). Mean platelet volume in nasal polyps. West Indian Med J.

[24] Duman TT, Aktas G, Atak B, Kocak MZ (2018). Bolu Abant Izzet Baysal University Hospital DOIM: Is Mean Platelet Volume to Platelet ratio a promising indicator of diabetic regulation in type 2 diabetes mellitus?. The Journal of Medical Research.

[25] Aktas G, Alcelik A, Tekce BK, Tekelioglu V, Sit M, Savli H (2014). Red cell distribution width and mean platelet volume in patients with irritable bowel syndrome. Prz Gastroenterol.

[26] Sit M, Aktas G, Ozer B, Kocak MZ, Erkus E, Erkol H, Yaman S, Savli H (2019). Mean platelet volume: an overlooked herald of malignant thyroid nodules. ACTA CLIN CROAT.

[27] Aktas G, Kocak MZ, TaslamaciogluDuman T, Erkus E, Atak BM, Sit M, Savli H (2018). Mean Platelet Volume (MPV) as an inflammatory marker in type 2 diabetes mellitus and obesity. Bali medical Journal.

[28] Coskun ME, Alidris A, Temel MT, Akbayram S, Hizli S (2019). Plateletcrit: A possible biomarker of inflammation in hepatitis A infection. NIGER J CLIN PRACT.

[29] Wang J, Zhou D, Dai Z, Li X (2021). Association Between Systemic Immune-Inflammation Index and Diabetic Depression. CLIN INTERV AGING.

[30] Walzik D, Joisten N, Zacher J, Zimmer P (2021). Transferring clinically established immune inflammation markers into exercise physiology: focus on neutrophil-to-lymphocyte ratio, platelet-to-lymphocyte ratio and systemic immune-inflammation index. EUR J APPL PHYSIOL.

[31] Atak B, Aktas G, Duman TT, Erkus E, Kocak MZ, Savli H (2019). Diabetes control could through platelet-to-lymphocyte ratio in hemograms. Rev Assoc Med Bras (1992).

[32] Arora S, Patro S, Nath P, Arora S (2020). Red cell distribution width(RDW) to platelet ratio (RPR): A novel marker in early prediction of severity of Acute Pancreatitis. J Assoc Physicians India.

[33] Bahrami A, Khorasanchi Z, Sadeghnia HR, Tayefi M, Avan A, Ferns GA, Bahrami-Taghanaki H, Ghayour-Mobarhan M (2019). Depression in adolescent girls: Relationship to serum vitamins a and E, immune response to heat shock protein 27 and systemic inflammation. J Affect Disord.

[34] Erkus E, Duman TT, Kocak MZ, Kosekli MA, Atak BM (2018). Mean Platelet volume to platelet and red cell distribution width to platelet ratios in Irritable Bowel Syndrome. Experimental Biomedical Research.

[35] Karagoz I, Öztürk H (2019). Does hemogram biomarkers predict mortality in intensive care population. Experimental Biomedical Research.

[36] Guo M, Sun T, Zhao Z, Ming L (2021). Preoperative Platelet to Albumin Ratio Predicts Outcome of Patients with Non-Small-Cell Lung Cancer. Ann Thorac Cardiovasc Surg.

[37] Osimo EF, Cardinal RN, Jones PB, Khandaker GM (2018). Prevalence and correlates of low-grade systemic inflammation in adult psychiatric inpatients: An electronic health record-based study. Psychoneuroendocrino.

[38] Zazula R, Dodd S, Dean OM, Berk M, Bortolasci CC, Verri WJ, Vargas HO, Nunes S (2022). Cognition-immune interactions between executive function and working memory, tumour necrosis factor-alpha (TNF-alpha) and soluble TNF receptors (sTNFR1 and sTNFR2) in bipolar disorder. World J Biol Psychiatry.

[39] Maffioletti E, Minelli A, Tardito D, Gennarelli M (2020). Blues in the brain and beyond: molecular bases of major depressive disorder and relative pharmacological and non-pharmacological treatments. Genes (Basel).

[40] Leonard BE (2001). The immune system, depression and the action of antidepressants. Prog Neuropsychopharmacol Biol Psychiatry.

[41] Wysokinski A, Szczepocka E (2016). Platelet parameters (PLT, MPV, P-LCR) in patients with schizophrenia, unipolar depression and bipolar disorder. Psychiatry Res.

[42] Fusar-Poli L, Natale A, Amerio A, Cimpoesu P, Grimaldi FP, Aguglia E, Amore M, Serafini G, Aguglia A (2021). Neutrophil-to-Lymphocyte, Platelet-to-Lymphocyte and Monocyte-to-Lymphocyte Ratio in Bipolar Disorder. Brain Sci.

[43] Ozdin S, Usta MB (2021). A comparison of inflammatory markers in manic and euthymic states of bipolar disorder. Nord J Psychiatry.

[44] Inanli I, Aydin M, Caliskan AM, Eren I (2019). Neutrophil/lymphocyte ratio, monocyte/lymphocyte ratio, and mean platelet volume as systemic inflammatory markers in different states of bipolar disorder. Nord J Psychiatry.

[45] Mazza MG, Tringali A, Rossetti A, Botti RE, Clerici M (2019). Cross-sectional study of neutrophil-lymphocyte, platelet-lymphocyte and monocyte-lymphocyte ratios in mood disorders. Gen Hosp Psychiatry.

[46] Ataoglu A, Canan F (2009). Mean platelet volume in patients with major depression: effect of escitalopram treatment. J Clin Psychopharmacol.

[47] Mert DG, Terzi H (2016). Mean platelet volume in bipolar disorder: the search for an ideal biomarker. Neuropsychiatr Dis Treat.

[48] Aleksovski B, Neceva V, Vujovic V, Manusheva N, Rendevski V, Novotni A, Filipce A, Spasovska A, Sofijanova A, Aleksovski V (2018). SSRI-reduced platelet reactivity in non-responding patients with life-long Recurrent Depressive Disorder: Detection and involved mechanisms. THROMB RES.

[49] Cai L, Xu L, Wei L, Chen W (2017). Relationship of mean platelet volume to MDD: a retrospective study. Shanghai Arch Psychiatry.

[50] Yu Q, Weng W, Zhou H, Tang Y, Ding S, Huang K, Liu Y (2020). Elevated platelet parameter in first-episode schizophrenia patients: a cross-sectional study. J Interferon Cytokine Res.

[51] Kounis NG, Koniari I, Plotas P, Soufras GD, Tsigkas G, Davlouros P, Hahalis G (2021). Inflammation, Thrombosis, and Platelet-to-Lymphocyte Ratio in Acute Coronary Syndromes. Angiology.

[52] Zhang Y, Zheng L, Quan L, Du L (2021). Prognostic role of platelet-to-lymphocyte ratio in oral cancer: A meta-analysis. J Oral Pathol Med.

[53] Mazza MG, Lucchi S, Tringali A, Rossetti A, Botti ER, Clerici M (2018). Neutrophil/lymphocyte ratio and platelet/lymphocyte ratio in mood disorders: A meta-analysis. Prog Neuropsychopharmacol Biol Psychiatry.

[54] Aguglia A, Amerio A, Asaro P, Caprino M, Conigliaro C, Giacomini G, Parisi VM, Trabucco A, Amore M, Serafini G (2021). High-lethality of suicide attempts associated with platelet to lymphocyte ratio and mean platelet volume in psychiatric inpatient setting. World J Biol Psychiatry.

[55] Djordjevic D, Rondovic G, Surbatovic M, Stanojevic I, Udovicic I, Andjelic T, Zeba S, Milosavljevic S, Stankovic N, Abazovic D (2018). Neutrophil-to-Lymphocyte Ratio, Monocyte-to-Lymphocyte Ratio, Platelet-to-Lymphocyte Ratio, and Mean Platelet Volume-to-Platelet Count Ratio as Biomarkers in Critically Ill and Injured Patients: Which Ratio to Choose to Predict Outcome and Nature of Bacteremia?. Mediators Inflamm.

[56] Ritchie RF, Palomaki GE, Neveux LM, Navolotskaia O, Ledue TB, Craig WY (1999). Reference distributions for the negative acute-phase serum proteins, albumin, transferrin and transthyretin: a practical, simple and clinically relevant approach in a large cohort. J Clin Lab Anal.

[57] Balcioglu YH, Kirlioglu SS (2020). C-Reactive Protein/Albumin and Neutrophil/Albumin Ratios as Novel Inflammatory Markers in Patients with Schizophrenia. Psychiatry Investig.

[58] Liu T, Zhong S, Liao X, Chen J, He T, Lai S, Jia Y (2015). A Meta-Analysis of Oxidative Stress Markers in Depression. PloS One..

[59] Kok KB, Unalan OP, Yuksel OO, Sozen S, Cihnioglu R, Kalelioglu T, Ilnem MC, Karamustafalioglu N (2020). Resolvin D1 as a novel anti-inflammatory marker in manic, depressive and euthymic states of bipolar disorder. Nord J Psychiatry.

[60] Bartoli F, Crocamo C, Dakanalis A, Brosio E, Miotto A, Capuzzi E, Clerici M, Carra G (2017). Purinergic system dysfunctions in subjects with bipolar disorder: A comparative cross-sectional study. Compr Psychiatry.

